# The Gastric CB1 Receptor Modulates Ghrelin Production through the mTOR Pathway to Regulate Food Intake

**DOI:** 10.1371/journal.pone.0080339

**Published:** 2013-11-26

**Authors:** Lucia L. Senin, Omar Al-Massadi, Cintia Folgueira, Cecilia Castelao, Maria Pardo, Silvia Barja-Fernandez, Arturo Roca-Rivada, Maria Amil, Ana B. Crujeiras, Tomas Garcia-Caballero, Enrico Gabellieri, Rosaura Leis, Carlos Dieguez, Uberto Pagotto, Felipe F. Casanueva, Luisa M. Seoane

**Affiliations:** 1 Grupo Fisiopatologia Endocrina, Instituto de Investigacion Sanitaria de Santiago de Compostela (IDIS), Complexo Hospitalario Universitario de Santiago (CHUS/SERGAS), Santiago de Compostela, Spain; 2 CIBER Fisiopatologia Obesidad y Nutricion (CB06/03), Instituto de Salud Carlos III, Santiago de Compostela, Spain; 3 Department of Physiology, Research Centre of Molecular Medicine and Chronic Diseases (CIMUS), Instituto de Investigacion Sanitaria Santiago de Compostela (IDIS). Santiago de Compostela, Spain; 4 Grupo Obesidomica, Instituto de Investigacion Sanitaria de Santiago de Compostela (IDIS), Complexo Hospitalario Universitario de Santiago (CHUS/SERGAS), Santiago de Compostela, Spain; 5 Laboratorio de Endocrinologia Molecular y Celular, Instituto de Investigación Sanitaria de Santiago (IDIS), Complejo Hospitalario de Santiago (CHUS/SERGAS), Santiago de Compostela, Spain; 6 Departamento de Ciencias Morfologicas, Facultad de Medicina. USC. Complejo Hospitalario de Santiago (CHUS/SERGAS), Santiago de Compostela, Spain; 7 Department of Translational Medicine, Amedeo Avogadro University, Novara, Italy; 8 Unit of Investigation in Nutrition, Growth and Human Development of Galicia, Pediatric Department (USC). Instituto de Investigación Sanitaria de Santiago de Compostela (IDIS), Complexo Hospitalario Universitario de Santiago, Santiago de Compostela, Spain; 9 Endocrinology Unit and Center for Applied Biomedical Research, Department of Medical and Surgical Sciences, Hospital S.Orsola-Malpighi, Alma Mater University of Bologna, Bologna, Italy; Universidad Miguel Hernández de Elche, Spain

## Abstract

Over the years, the knowledge regarding the relevance of the cannabinoid system to the regulation of metabolism has grown steadily. A central interaction between the cannabinoid system and ghrelin has been suggested to regulate food intake. Although the stomach is the main source of ghrelin and CB1 receptor expression in the stomach has been described, little information is available regarding the possible interaction between the gastric cannabinoid and ghrelin systems in the integrated control of energy homeostasis. The main objective of the present work was to assess the functional interaction between these two systems in terms of food intake using a combination of in vivo and in vitro approaches. The present work demonstrates that the peripheral blockade of the CB1 receptor by rimonabant treatment decreased food intake but only in food-deprived animals. This anorexigenic effect is likely a consequence of decreases in gastric ghrelin secretion induced by the activation of the mTOR/S6K1 intracellular pathway in the stomach following treatment with rimonabant. In support of this supposition, animals in which the mTOR/S6K1 intracellular pathway was blocked by chronic rapamycin treatment, rimonabant had no effect on ghrelin secretion. Vagal communication may also be involved because rimonabant treatment was no longer effective when administered to animals that had undergone surgical vagotomy. In conclusion, to the best of our knowledge, the present work is the first to describe a CB1 receptor-mediated mechanism that influences gastric ghrelin secretion and food intake through the mTOR pathway.

## Introduction

The stimulatory effect of *Cannabis sativa* on appetite has been well known for centuries [1]. In recent years, the characterization of the specific cannabinoid CB1 and CB2 receptors and the isolation of endogenous cannabinoids have revealed the existence of an endocannabinoid system. The scientific community has become increasingly interested in the implications of this system for body weight regulation; nevertheless, the mechanisms behind the relationship between this system and body weight regulation are still not well characterized [2].

Knowledge about energy homeostasis regulation was boosted with the isolation of ghrelin from the stomach in 1999 [3]; and this gastric-derived peptide has been proposed to be a link between the stomach and the central nervous system. The interaction between ghrelin and the cannabinoid system has previously been proven via the demonstration of the inhibitory effect of centrally and peripheral administered rimonabant (an antagonist of the CB1 receptor) on the orexigenic and GH releasing effect of ghrelin [4]–[6]. Additionally, it has been reported that both systems depend on interactions with the AMPK pathway in the hypothalamus and peripheral tissues [7], [8]. Finally, the counteraction of peripheral CB1 receptor antagonism on ghrelin orexigenic action has been described [9]; however, the mechanism behind that interaction has not been elucidated. Traditionally, the regulation of appetite has been attributed to the CB1 cannabinoid receptors located in the brain [10]. However, a functional interaction between endocannabinoid and ghrelinergic systems might be hypothesized to occur in the gastrointestinal tract [11]. This hypothesis is based on the expression of CB1 receptors in the epithelium of gastric mucosa, primarily in the fundus of the stomach where ghrelin is synthesized and secreted [12]. In support of this, it was observed that CB1 cannabinoid antagonists such as rimonabant have no effect when directly injected into the brains of food-deprived animals, whereas systemically administered cannabinoid agents affect food intake [13], [14].

In this context, the hypothesis of the present work is that a gastric mechanism regulating food intake that depends on the nutritional status of the animal and is dependent on an interaction between the cannabinoid system and ghrelin exists. Furthermore, we postulated that this interaction may be mediated by mTOR (mammalian target of rapamycin); mTOR is an energy sensor that is a component of at least two multi-protein complexes: mTOR complex 1 (mTORC1) and mTOR complex 2 (mTORC2). mTORC1 phosphorylates and modulates the activity of the serine/threonine ribosomal protein S6 kinase 1 (S6K1), which, in turn, phosphorilates and activate S6, a ribosomal protein involved in translation [15]–[17].

## Materials and Methods

### Ethics Statement

The authors of this manuscript declare that the animal work in this study was approved by the Animal Care Committee of Santiago de Compostela University (Santiago de Compostela, Spain) in accordance with our institutional guidelines and the European Union standards for the care and use of experimental animals.

### Animal and experimental designs

Sprague-Dawley rats were used. Rats were housed for all experiments, rats were housed in air-conditioned rooms (22–24°C) under a controlled light/dark cycle (12 hours light, 12 hours darkness) with free access to food and water (n = 8–10). The surgical procedures were performed under anesthesia induced by intraperitoneal (ip) injection of a mixture of ketamine and xylazine (ketamine 100 mg/Kg body weight + xylazine 15 mg/kg body weight). The animals were euthanatized by decapitation. Trunk blood was collected and immediately centrifuged, and plasma was stored at –80°C for the biochemical measurements.

### Experiment 1: Food intake studies

Adult male rats weighing 250–300 g were implanted with a chronic intracerebroventricular (*icv)* cannula as described below [18]. The amount of food ingested daily by each rat was measured on days prior to the experiment.

One group of rats had food available *ad libitum* (fed rats). The second group was food-deprived for 12 hours before the experiment (nocturnally fasted rats), and the third group was subjected to surgical vagotomy one week before the experiment and food-deprived for 12 hours before the experiment (nocturnally fasted vagotomy rats). Each of the 3 groups was further divided into the following 4 treatments (n = 8–10): vehicle ip (a solution of saline-DMSO 70%) plus vehicle icv (saline), vehicle ip (a solution of saline-DMSO 70%) plus human ghrelin (Global Peptides, Fort Collins, Colorado, USA) (6 µg/rat icv), rimonabant (3 mg/kg ip) plus vehicle icv (saline), and rimonabant (3 mg/kg ip) plus human ghrelin (6 µg/rat icv). Total food intake was assessed at 1, 2, 4 and 6 hours post-injection.

Statistical analyses were performed, and comparisons between groups were conducted using the Mann-Whitney non-parametric test.

### Experiment 2: Immunohistochemical studies of the location of ghrelin and CB1 in gastric tissue from *ad libitum* fed rats

Immunohistochemistry was performed on sections obtained from the stomach of the *ad libitum* fed animals. Immunopositivies for the CB1 receptor and ghrelin in gastric tissue were examined. Immunoreactivity for synaptophysin (a marker of neuroendocrine cells) was also examined. Additionally, the corresponding negative controls without primary antibodies were performed.

### Experiment 3: Variations in plasma ghrelin and gastric ghrelin secretion in animals with CB1 pharmacological blockade

**3.a. In vivo experiments.** To study whether peripheral block of the endogenous cannabinoid system directly affected ghrelin secretion by the stomach and plasma ghrelin levels, adult male rats were assigned to one of eight experimental groups. The *ad libitum* fed group received intraperitoneal (ip) injection of rimonabant (3 mg/kg) (rimonabant) or vehicle (a solution of saline-DMSO 70%) (Control) 1 hour before the euthanasia, and the *ad libitum* animals subjected to surgical vagotomy 1 week before the experiment were treated with rimonabant ip (3 mg/kg) (Vagotomy rimonabant) or vehicle (a solution of saline-DMSO 70%, i.e., the Vagotomy group) and euthanatized 1 hour later. The other four groups received the treatments described above and were fasted for 36 hours.

Upon decapitation, gastric tissue explants obtained from the experimental models were incubated as described below [19], and gastric ghrelin secretion was measured by radioimmunoassay (RIA) in the culture medium. Additionally, trunk blood was collected, and ghrelin levels were measured in the plasma with a commercial kit for radioimmunoassay.

**3.b. In vitro experiments.** Explants of gastric tissue obtained from the *ad libitum*/fasted adult male rats receiving sham operations or vagotomies (the method described below), were incubated for 3 h with rimonabant (1 µM) or vehicle. The medium was collected and ghrelin levels in the culture medium were measured by RIA.

Two groups of gastric explants from fasting animals were incubated for 3 h with 2.5 and 5 µM doses of rimonabant.

### Experiment 4: Variations in the levels of expression of ghrelin and CB1 in gastric mucosae

The variations in ghrelin and CB1 mRNA levels in the gastric mucosae obtained from previously described experimental groups were examined with real-time PCR. Additionally, ghrelin peptide contents in the gastric mucosae obtained from the animal groups listed in Experiment 2a were assayed by Western blotting.

### Experiment 5: The effects of pharmacological blockade of CB1 on the regulation of gastric mTOR signaling

The modulation of gastric mTOR signaling by the peripheral blockade of the endogenous cannabinoid system was studied. Adult male rats were assigned to one of the following experimental groups: 36-hour fasted animals that received vehicle (a solution of saline-DMSO 70% ip for 6 days) (control), 36-hour fasted animals that received vehicle (a solution of saline-DMSO 70% ip for 6 days) and were treated ip with rimonabant (3 mg/kg) 1 hour before euthanasia (rimonabant), 36-hour fasted animals treated with rapamycin (1 mg/Kg ip for 6 days) (rapamycin) and 36-hour fasted animals that were treated with rapamycin (1 mg/Kg ip for 6 days) and ip rimonabant (3 mg/kg) 1 hour before the euthanasia (rapamycin-rimonabant). Rapamycin was purchased from Santa Cruz Biotechnology, Inc. (Santa Cruz, CA, USA).

The pmTOR and pS6K1 protein contents of the gastric mucosae of the animals of these experimental groups were assayed by Western blot and standardized against the measures of mTOR and S6K1, respectively.

### Experiment 6: Effects of pharmacological blockade of CB1 on plasma ghrelin, gastric ghrelin secretion and ghrelin mRNA levels in animals with rapamycin treatment-induced inhibition of gastric mTOR signaling

The involvement of gastric mTOR signaling in the effect of the peripheral blockade of the endogenous cannabinoid system on gastric and plasma ghrelin regulation was studied. Adult male rats were assigned to one of the following experimental groups: 36-hour fasted animals (Control), 36-hour fasted animals treated with rimonabant (3 mg/kg) 1 hour before euthanasia (Rimonabant), 36-hour fasted animals treated with rapamycin (1 mg/Kg ip for 6 days) (Rapamycin) and, 36-hour fasted animals treated with rapamycin (1 mg/Kg ip for 6 days) and ip rimonabant (3 mg/kg) 1 hour before euthanasia (Rapamycin-Rimonabant).

Gastric ghrelin secretion from gastric explants and plasma ghrelin levels were measured by RIA in each of the experimental groups. Ghrelin mRNA levels in the gastric mucosae were analyzed with real-time PCR.

### Experiment 7: The effects of pharmacological blockade of CB1 with AM281 on plasma ghrelin, and gastric ghrelin secretion

To confirm the results obtained with rimonabant, a group of fasted animals was treated with AM-281 (Tocris Cookson Inc., Ellisville, MO), which is a CB1 antagonist that is different from rimonabant. Adult male rats were assigned to one of 2 experimental groups: the first group of rats were fasted for 36 hours and received an intraperitoneal (ip) injection of AM281 (3 mg/kg) 1 hour before the euthanasia (AM281), the second group was fasted for 36 hours and received an injection of vehicle (DMSO), 1 hour before euthanasia (Control). Upon decapitation, gastric tissue explants obtained from the experimental animals were incubated as described below [19], and gastric ghrelin secretion was measured by radioimmunoassay (RIA) with a commercial kit (Phoenix Pharmaceuticals Inc, USA) in the culture medium. Additionally, trunk blood was collected, and plasma ghrelin levels were measured with the commercial radioimmunoassay kit.

### Experiment 8: Pharmacological blockade of CB1 with AM281 in the regulation of gastric mTOR signaling

pmTOR and pS6K1 protein contents in the gastric mucosae of the animal groups described in experiment 7 were assayed by Western blot and standardized to the measures of mTOR and S6K1, respectively.

### Experimental techniques

**Tissue explant culture.** Tissue explants were obtained from adult Sprague-Dawley rats. After euthanasia, the stomach was rapidly excised and transported to the lab in Krebs-Ringer-HEPES buffer (NaCl, 125 mmol/l; KCl, 5 mmol/l; MgSO4, 1.2 mmol/l; KH2PO4, 1.3 mmol/l; CaCL2, 2 mmol/l; glucose, 6 mmol/l; HEPES 25 mmol/l; pH = 7.4). After removing the blood vessels and connective tissue, the stomach tissue was washed with sterile Krebs-Ringer-HEPES buffer. The tissue explants were placed in six-well dishes containing 2.5 ml of Dulbeccós modified Eaglés medium supplemented with L-glutamine (200 mM), penicillin (1000 U/ml) and streptomycin (100 µg/ml). After a pre-incubation period of 1 hour at 37°C under a humidified atmosphere of 95% air–5% CO2, the media were aspirated, and 2.5 ml of fresh medium was dispensed into each well. The culture medium was then collected after 2 hours. The samples were stored at –80°C until the ghrelin assay. The ghrelin levels were measured by RIA using commercial kits (rat ghrelin RIA, Millipore Darmstadt, Germany) [20].

### Western-blot studies

Whole tissue proteins were prepared by homogenization using a TissueLyser II (Qiagen, Tokyo, Japan) in cold RIPA buffer [containing 200 mM Tris/HCl (pH 7.4), 130 mM NaCl, 10%(v/v) glycerol, 0.1%(v/v) SDS, 1%(v/v) Triton X-100, 10 mM MgCl2] with anti-proteases and anti-phosphatases (Sigma-Aldrich;St.Louis, MO). The tissue lysates were centrifuged for 10 minutes at 18000 g in a microfuge at 4°C. Next, equal amounts of protein (10 or 50 µg/well) were run on sodium–dodecyl sulfate-polyacrylamide gels (SDS–PAGE) and electroblotted onto nitrocellulose membranes. The membranes were probed successively with primary antibodies and peroxide-conjugated secondary antibodies (Thermo Scientific, Pierce, Rockford, IL, USA). Specific antigen-antibody bindings were visualized using a chemiluminescence method according to the manufacturer’s instructions (Super Signal West Dura Extended Duration Substrate, Thermo Scientific, Pierce). Primary anti-ghrelin (C-18) was purchased from Santa Cruz Biotechnology (CA, USA). Anti-β-actin (A-5316, Sigma Chemical Co., St Louis, MO, USA), anti-pmTOR (s2448), anti-mTOR, anti-S6k1 and anti-pS6K1 (Thr389) antibodies from Cell Signaling Technology (MA, USA) were also used.

Western blots were performed using independent samples from different rats from each group [20]. β-actin detection was performed for all western blots as a loading control. The Western blot data are expressed as the means±SEM of the percentages normalized to β-actin levels (arbitrary units). Data analyses were conducted using GraphPad Prism 5 software and the Mann-Whitney U test and significance is indicated as follows: *p<0.05 and **p<0.01.

### RNA isolation and real-time quantitative RT-PCR

Total RNA was isolated from the stomach mucosae of animals using TRIzol (Invitrogen, CA, USA) according to the manufacturer’s recommendations. The extracted total RNA was purified with DNase treatment using a DNA-free kit as a template (Ambion, USA) to generate first-strand cDNAs using a High-Capacity cDNA Reverse Transcription kit (Applied Biosystems, USA). Quantitative real-time PCR was performed using a StepOne Plus instrument (Applied Biosystems) with specific Taqman qRT-PCR primers and probes (Table 1). For analyses, the ghrelin gene expression levels were normalized to the expression mRNA levels of the housekeeping gene hypoxanthine phosphoribosyltransferase 1 (HPRT_1_) (TaqMan: Applied Biosystems) and are expressed relative to the average value of the control group.

**Table 1:** Primers for the real time qPCR analyses.

Gene symbol	Gene description	TaqMan gene expression assay number	GenBank accession number
HPRT	hypoxanthinephosphoribosyltransferase 1 (REFERENCE GENE)	Rn01527840_m1	NM_012583.2
GHRL	ghrelin/obestatin prepropeptide	Rn01425835_m1	NM_021669.2
CNR1/CB1	cannabinoid receptor 1 (brain)	Rn00562880_m1	NM_012784.4

### Immunohistochemical studies

The gastric wall samples were immersion fixed in 10% neutral buffered formalin for 24 h and embedded in paraffin. Four millimeter thick sections were mounted on FLEX IHC microscope slides (Dako, Glostrup, Denmark) and heated in an oven at 60°C for 1 h. Immunohistochemical analyses was automatically performed using an AutostainerLink 48 (Dako). After deparaffinization and epitope retrieval in EnVision FLEX target retrieval solution (low pH) for 20 min at 97°C, the slides were allowed to cool in PT Link to 65°C and then in Dako wash buffer for 5 min at room temperature. The immunostaining protocol included the following: (1) incubation in EnVision FLEX peroxidase-blocking reagent (Dako) for 5 min; (2) incubation with ghrelin (C-18) goat polyclonal antibody raised against a peptide mapping to an internal region of human-origin ghrelin (Santa Cruz Biotechnology, Santa Cruz, CA) at a dilution of 1/500 or ready to use synaptophysin mouse monoclonal antibody Clone SY38 (Dako) or CB1 (H-150) rabbit polyclonal antibody raised against amino acids 1–50 mapping to the N-terminus of human-origin CB1 (Santa Cruz Biotechnology), all for 30 min; and (3) goat antibody to ghrelin, which involved an LSAB+ System-HRP consisting of incubation with a universal biotinylated link (15 min) and streptavidin-HRP (15 min), and for the rabbit antibody to CB1 and mouse antibody to synaptophysin an,incubation with the EnVision FLEX/HRP (dextran polymer conjugated with horseradish peroxidase and affinity-isolated goat anti-mouse and anti-rabbit immunoglobulins) were used (20 min). In addition, there was an incubation in (4) substrate working solution (mix) (3,3’-diaminobenzidine tetrahydrochloridechromogen solution) (Dako) for 10 min and (5) EnVision FLEX hematoxylin (Dako) for 9 min.

### Biochemical analysis

Total ghrelin levels were determined via double-antibody RIA using reagents kits and methods provided by Millipore (Darmstadt, Germany). The samples for measuring the secretion from the tissue explants were obtained directly by collecting the culture medium. The samples were analyzed by RIA as previously described. The assay sensitivity limit was 93 pg/ml. The results are expressed as pg/ml of total ghrelin per gram of tissue in the culture media.

The data are expressed as the means± SEMs and were assessed with Mann-Whitney U tests. *p<0.05 was considered significant.

### Surgical proceedings

**Implantation of intracerebroventricular cannula and ghrelin treatment.** Chronic intracerebroventricular (icv) cannulas were implanted under ketamine-xylazine anesthesia (50 mg/kg, i.p.) as described previously, their targeting of the lateral ventricle was confirmed with methylene blue staining [18]. The animals were caged individually and used for the experiments one week after surgery. During this postoperative recovery period, the rats became accustomed to the handling procedure under non-stressful conditions.

### Vagotomy

The surgical procedure was performed aseptically, and all surgical instruments were sterilized before use. The animals were operated under ketamine-xylazine anesthesia. The rats were placed on their backs and a midline abdominal incision was made. The liver was carefully moved to the right to expose the esophagus. The dorsal and ventral branches of the vagus nerve were exposed and dissected from the esophagus. Each branch of the nerve was ligated with surgical sutures at two points, which were as distal as possible to prevent bleeding, and cauterized between the sutures. The abdominal muscles and the skin were then sutured with surgical silk. Sham surgeries were also performed in which each trunk of the nerve was exposed but not tied or cauterized. One week after vagotomy, food intake measurements were performed as described previously. The effectiveness of the vagotomy was assessed by post-mortem stomach observation. Only the rats that exhibited increases in stomach size after the vagotomy were included in the analyses [21].

## Results

### Experiment 1: Ghrelin and cannabinoids interact to regulate food intake

**Figure 1:**
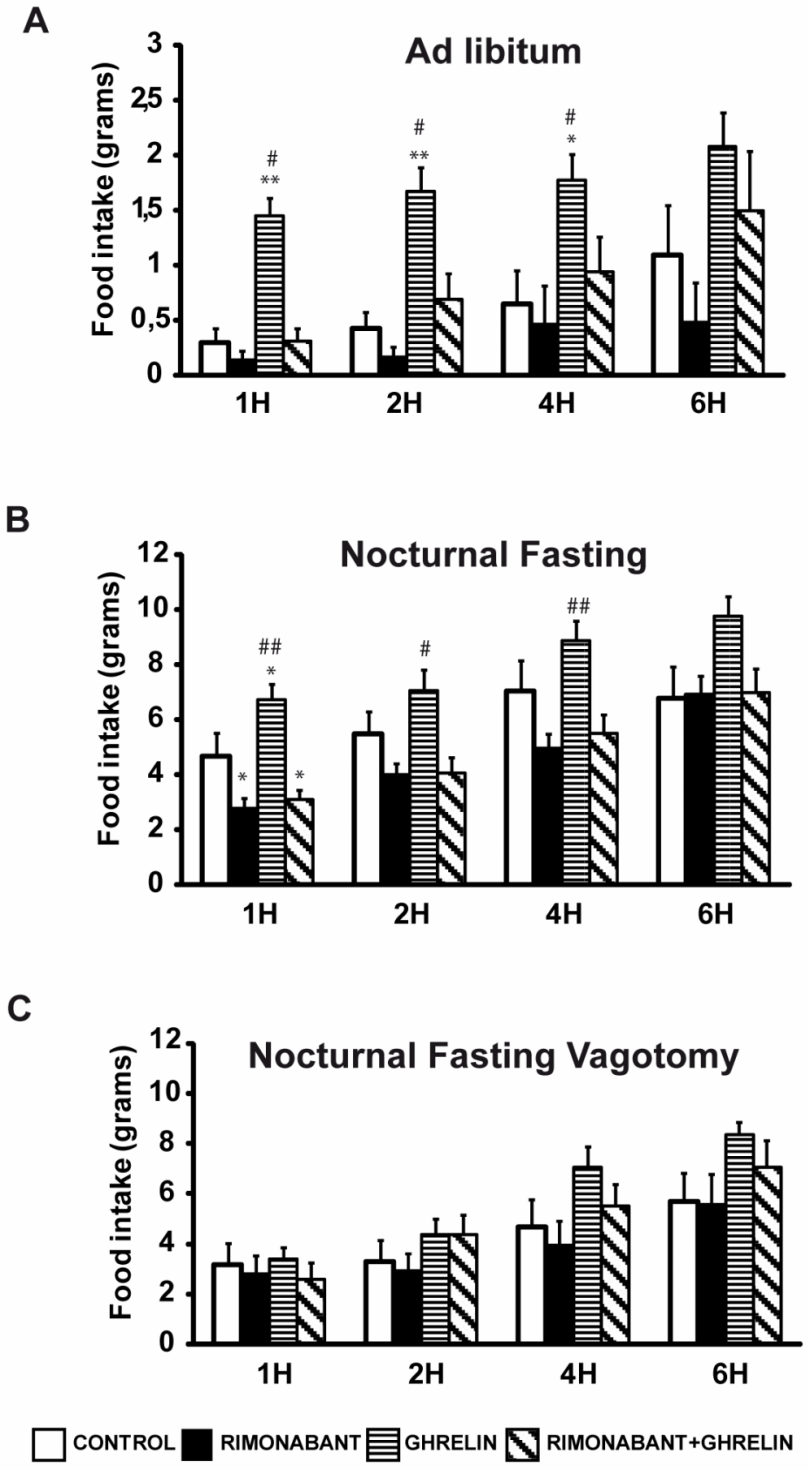
Food Intake measures in rats after icv treatment with ghrelin/vehicle (6 µg/rat) and ip treatment with rimonabant (3 mg/kg ip)/vehicle under different nutritional statuses: A) *Ad libitum* fed, B) nocturnal fasting, c) animals subjected to overnight fasting and previously subjected to surgical vagotomy. *vs control, ^#^vs. rimonabant+ghrelin.

Ghrelin injection induced increases in food intake at 1, 2 and 4 hours; these increases were more evident in the *ad libitum* group (control: 0.29±0.13, 0.42±0.15 and 0.65±0.30 vs ghrelin: 1.45±0.16, 1.66±0.22 and 1.77±0.24 grams of food intake at 1, 2 and 4 hours, resepectively; Figure 1A) than in the animals that were fasted overnight (control: 4.65±0.67, 5.46±0.79 and 7.02±0.79 vs ghrelin: 6.74±0.55, 7.05±0.77 and 8.88±0.72 grams of food intake, respectively; Figure 1B).

Peripheral treatment with rimonabant decreased basal food intake in the overnight fasted animals (Figure 1B; control: 4.65±0.67 vs rimonabant: 2.74±0.39 grams of food intake at 1 hour; p<0.05). However, this treatment had no effect on the satiated animals (control: 0.298±0.13 vs rimonabant: 0.128±0.09 grams of food intake; Figure 1A).

Ghrelińs orexigenic action was blocked by peripheral injections of rimonabant in both overnight fasted animals (ghrelin: 6.74±0.55 vs. ghrelin+rimonabant: 3.08±0.37 grams of food intake; p<0.01; Figure 1B) and satiated animals (ghrelin: 1.45±0.163 vs. ghrelin+rimonabant: 0.306±0.12 grams of food intake; p<0.01; Figure 1A).

Surgical vagotomy blocked the anorexigenic effects of rimonabant on both basal food intake and ghrelin-stimulated food intake (Figure 1C).

### Experiment 2: Immunohistochemical studies of the locations of ghrelin and CB1 receptors in gastric tissue from *ad libitum* fed rats

**Figure 2:**
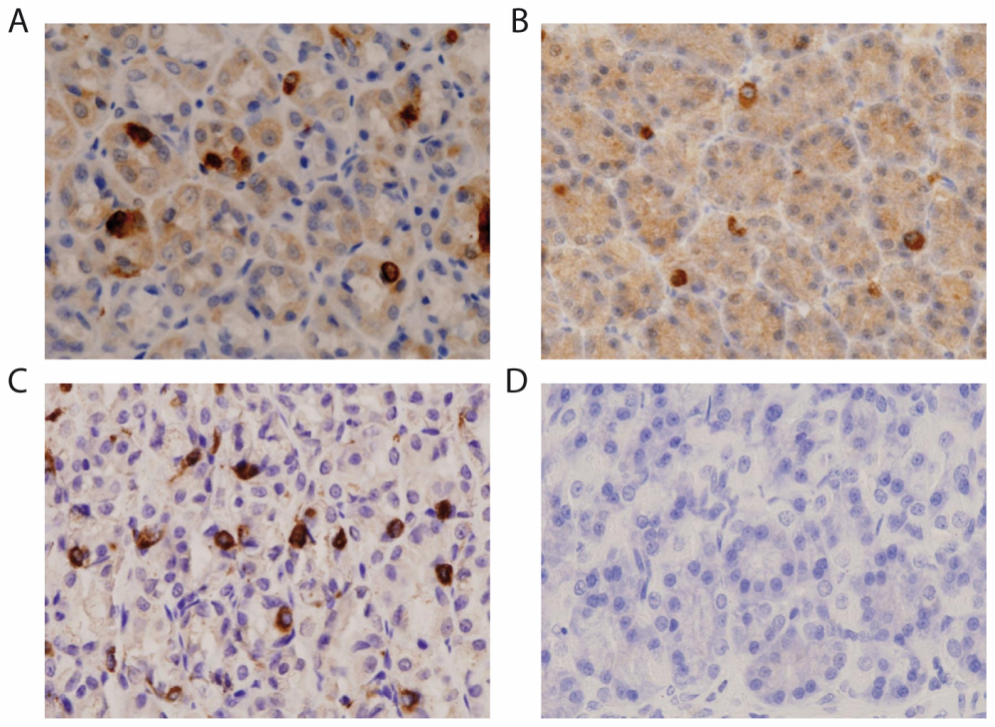
Immunohistochemical studies of the locatizations of ghrelin (A) and CB1 (B) in gastric tissue from *ad libitum* fed rats. Inmunoreactivities for both antibodies were found in isolated neuroendocrine cells primarily that were primary located at the bottom of the gastric glands. Labeling of neuroendocrine cells with synaptophysin is shown in panel (C). The corresponding negative control (D). Magnification 40x.

**Figure 3:**
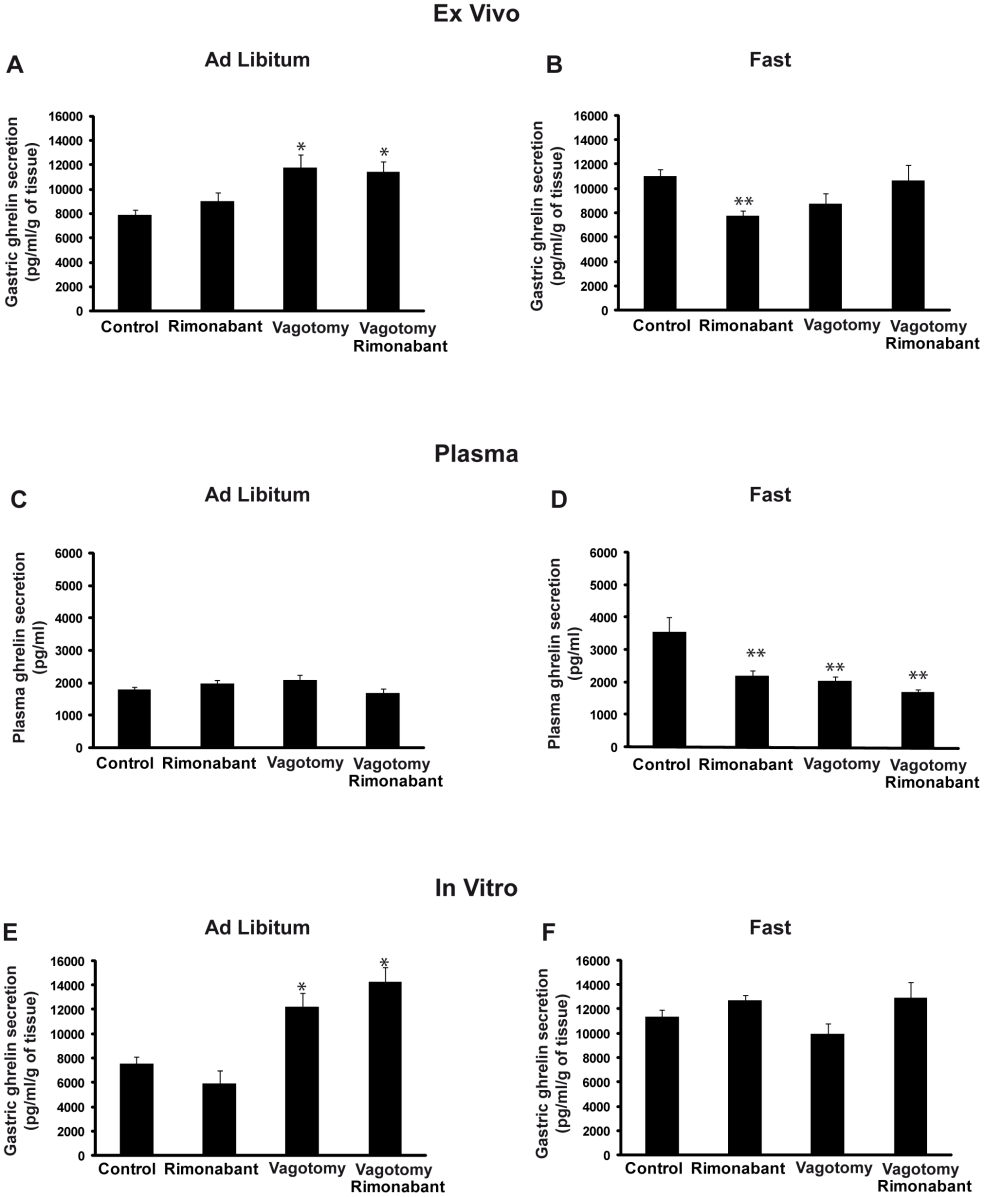
Gastric ghrelin secretion from tissue explants obtained from *ad libitum* fed animals (A) or 36-hour fasted animals (B) and plasma ghrelin levels from *ad libitum* fed animals (C) or 36-hour fasted animals (D) under different in vivo treatments (control/rimonabant), and surgical procedures (vagotomy/sham operated). Gastric ghrelin secretion from tissue explants from *ad libitum* fed animals (E) or 36-hour fasted animals (F) under different in vitro treatments (control/rimonabant) and surgical procedures (vagotomy/sham operated). *vs control.

To investigate whether CB1 receptor were present in the endocrine cells of the stomach, immunohistochemical analyses using specific antibodies for CB1 and ghrelin were performed on gastric fundus sections obtained from *ad libitum* fed rats. Immunoreactivities for ghrelin and CB1 were found in small dispersed cells situated primarily at the bottom of the gastric glands (Figure 2A and B). The size, morphology and distribution of these cells let us to identify them as neuroendocrine cells. Additionally, synaptohysin inmmunoreactivity confirmed the neuroendorine nature of these cells (Figure 3C). The negative control without CB1 or ghrelin antibodies is shown in Figure 2D.

### Experiment 3: Variations in plasma ghrelin and gastric ghrelin secretion in animals with CB1 pharmacological blockade

**3.a. In vivo.** Rimonabant treatment (ip) of *ad libitum* fed rats did not affect gastric ghrelin secretion, in either the sham operated rats or in vagotomized animals (Figure 3A). Surgical vagotomy increased gastric ghrelin secretion in the fed animals (fed: 7884.0±419 pg/ml/g tissue vs fed vagotomy: 11758±1043 pg/ml/g tissue; p<0.05). As expected, food restriction for 36 hours increased gastric ghrelin secretion with respect to the *ad libitum* animals (fed: 7884.0±419 pg/ml/g tissue vs fast: 11060.0±559.5 pg/ml/g tissue; p<0.01, Figure 3A and B). Surprisingly, rimonabant treatment of the fasted animals reversed the increased levels of gastric ghrelin secretion induced by the food deprivation (fast: 11060.0±559.5 pg/ml/g tissue vs. fast rimonabant: 7796±430.9 pg/ml/g tissue; p<0.01), and this effect was not observed in vagotomized animals subjected to food deprivation (fast vagotomy: 8749±832 pg/ml/g tissue vs fast vagotomy rimonabant: 10649±1314 pg/ml/g tissue; no significant difference, Figure 3B). Vagotomized animals in the fasting condition, exhibited a non-significant decrease in gastric ghrelin secretion compared to the control group (Figure 3B).

In keeping with the ex vivo data, rimonabant treatment did not affect circulating ghrelin levels in the *ad libitum* animals (Figure 3C). Increased circulating ghrelin levels were observed in the fasted animals relative to the *ad libitum* group (fed: 1797±73 vs fast: 3559±462 pg/ml; p<0.01) but were unaltered in the vagotomized animals (fed vagotomy: 1984±87.2 vs fast vagotomy: 2039±138 pg/ml) (Figure 3C and D). Similar to gastric ghrelin secretion, pharmacological cannabinoid receptor blockade prevented the food-deprivation induced increase in circulating ghrelin levels (fast: 3559±462 pg/ml vs. fast rimonabant: 2211±141 pg/ml; p<0.01) (Figure 3D).

**3b. Variation in ghrelin secretion from gastric explants in vitro treated with rimonabant.** Vagotomized animals exhibited increased gastric ghrelin secretion (fed: 7571±538 pg/ml/g tissue vs fed vagotomy: 12218±1115 pg/ml/g tissue; p<0.01; Figure 3E). Gastric explants from the fasted animals exhibited higher levels of ghrelin secretion than those from the *ad libitum* fed animals (fed: 7571±538 pg/ml/g tissue vs fast: 11394±901 pg/ml/g tissue) (Figure 3E and F). In contrast to the in vivo data, rimonabant directly added to the stomachs of food-deprived animals was not able to revert the increased levels of gastric ghrelin secretion induced by fasting (fast: 11394±901 pg/ml/g tissue vs fast rimonabant: 12739±2294 pg/ml/g tissue; no significant difference) (Figure 3F).

To reconfirm the lack of effect of rimonabant directly added to stomachs of food-deprived animals, two additional doses of rimonabant were tested (2.5 and 5 µM). These doses were not able to revert the increased levels of gastric ghrelin secretion induced by fasting (fast: 15351±1826pg/ml/g tissue vs fast rimonabant (2.5 µM): 10792±560 pg/ml/g tissue, fast rimonabant (5 µM): 13466±3050 pg/ml/g tissue; no significant difference) (Figure S1).

### Experiment 4: Variations in the expression levels of ghrelin and the CB1 receptor

**Figure 4:**
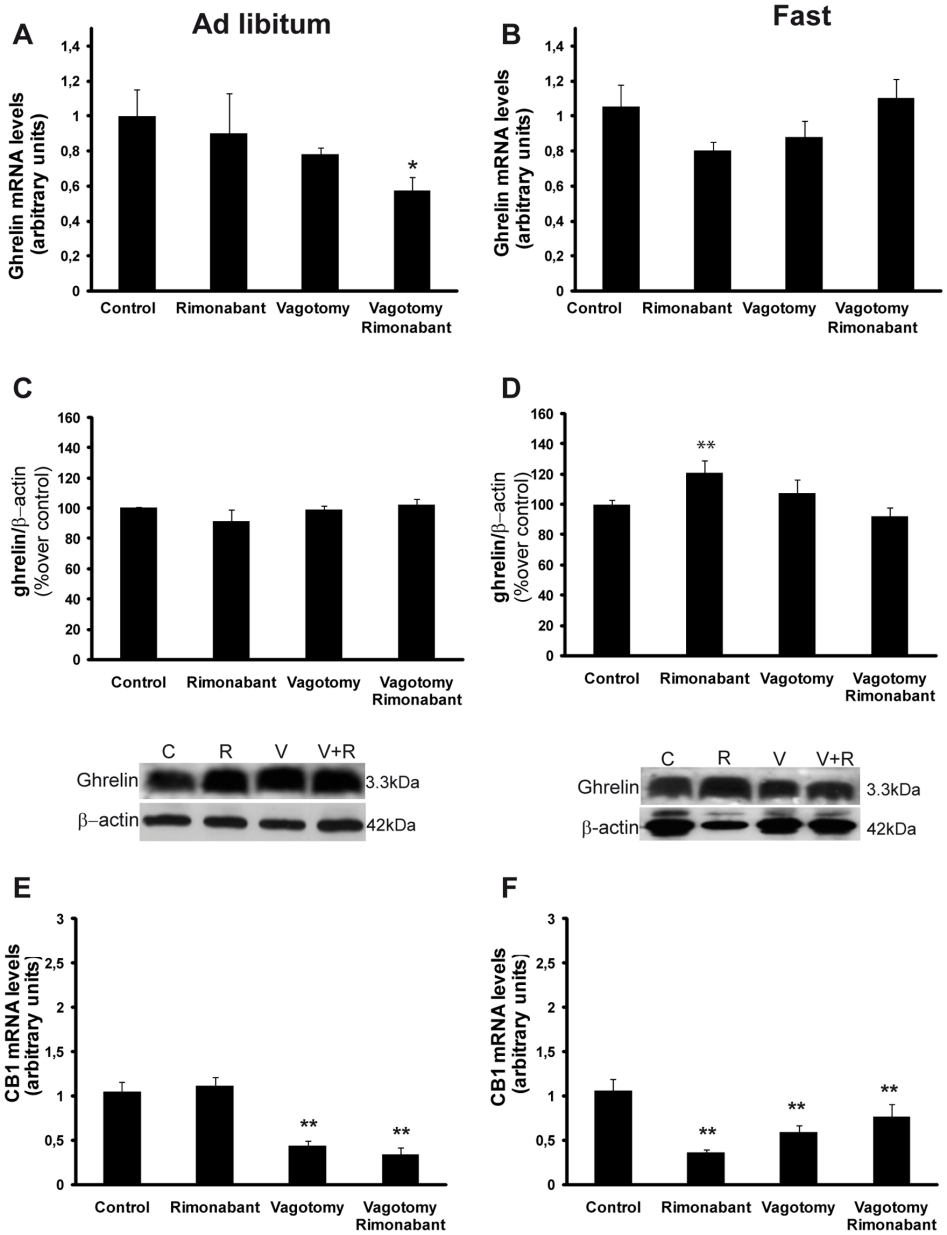
mRNA expression levels measured by real-time PCR for ghrelin in *ad libitum* fed animals (A) or 36-hour fasted animals (B). Ghrelin protein levels and representative Western blot from the mucosa from *ad libitum* fed animals (C) or 36-hours fasted animals (D). Animals that received different in vivo treatments (control/rimonabant) and surgical procedures (vagotomy/sham-operated). C: control, R: rimonabant, V: vagotomy; V+R: vagotomy+rimonabant. CB1 receptor mRNA levels in the gastric mucosae of *ad libitum* fed animals (E) or 36-hour fasted animals (F) and animals that received different in vivo treatments (control/rimonabant) and surgical procedures (vagotomy/sham operated). *vs control.

A decrease in ghrelin mRNA levels was observed in animals subjected to surgical vagotomy (fed: 1±0.15 arbitrary units vs fed vagotomy: 0.78±0.04 arbitrary units; fed rimonabant: 0.9±0.23 vs fed vagotomy rimonabant 0.57±0.08 arbitrary units; p<0.05) (Figure 4A).

Fasting for 36 hours did not affect the gastric ghrelin mRNA with respect to the *ad libitum* animals, although a slight tendency toward an increase in ghrelin mRNA was observed (fed: 1±0.15 vs fast: 1.05±0.13 arbitrary units; ns). However, rimonabant treatment (ip) of fasted animals slightly decreased ghrelin mRNA levels (fast: 1.05±0.13 arbitrary units vs. fast rimonabant: 0.8±0.05 arbitrary units), but this difference was not statistically significant (Figure 4B). Ghrelin mRNA levels were not changed in the vagotomized fasted animals (fast: 1.05±0.13 vs fast vagotomy: 1.1±0.11). Additionally, rimonabant treatment did not induce any effects in the animals subjected to surgical vagotomy (Figure 4B).

Rimonabant treatment of fasted animals increased the ghrelin contents of the stomachs (rimonabant: 120.68% vs control fast; p<0.01) (Figure 4D). Additionally, as shown in the representative western blot in Figure 4, the fasted animals exhibited lower stomach ghrelin contents compared to the fed animals (fast: 62.7% over control fed; p<0.01) (Figure 4C and D).

Rimonabant treatment (ip) of the *ad libitum* fed rats did not affect gastric CB1 mRNA levels in either the sham operated rats or the vagotomized animals (Figure 4E). However, vagotomy per se decreased CB1 mRNA expression (fed: 1.05±0.11 vs fed vagotomy: 0.44±0.05, p<0.01; fed vagotomy rimonabant: 0.34±0.05; p<0.01) (Figure 4E).

In contrast to ghrelin, CB1 mRNA levels decreased (Figure 4F) significantly after peripheral rimonabant treatment of fasted animals (fast: 1.06±0.13 vs fast rimonabant: 0.36±0.03 arbitrary units; p<0.01).

### Experiment 5: The effects of pharmacological blockade of CB1 on gastric mTOR signaling

**Figure 5:**
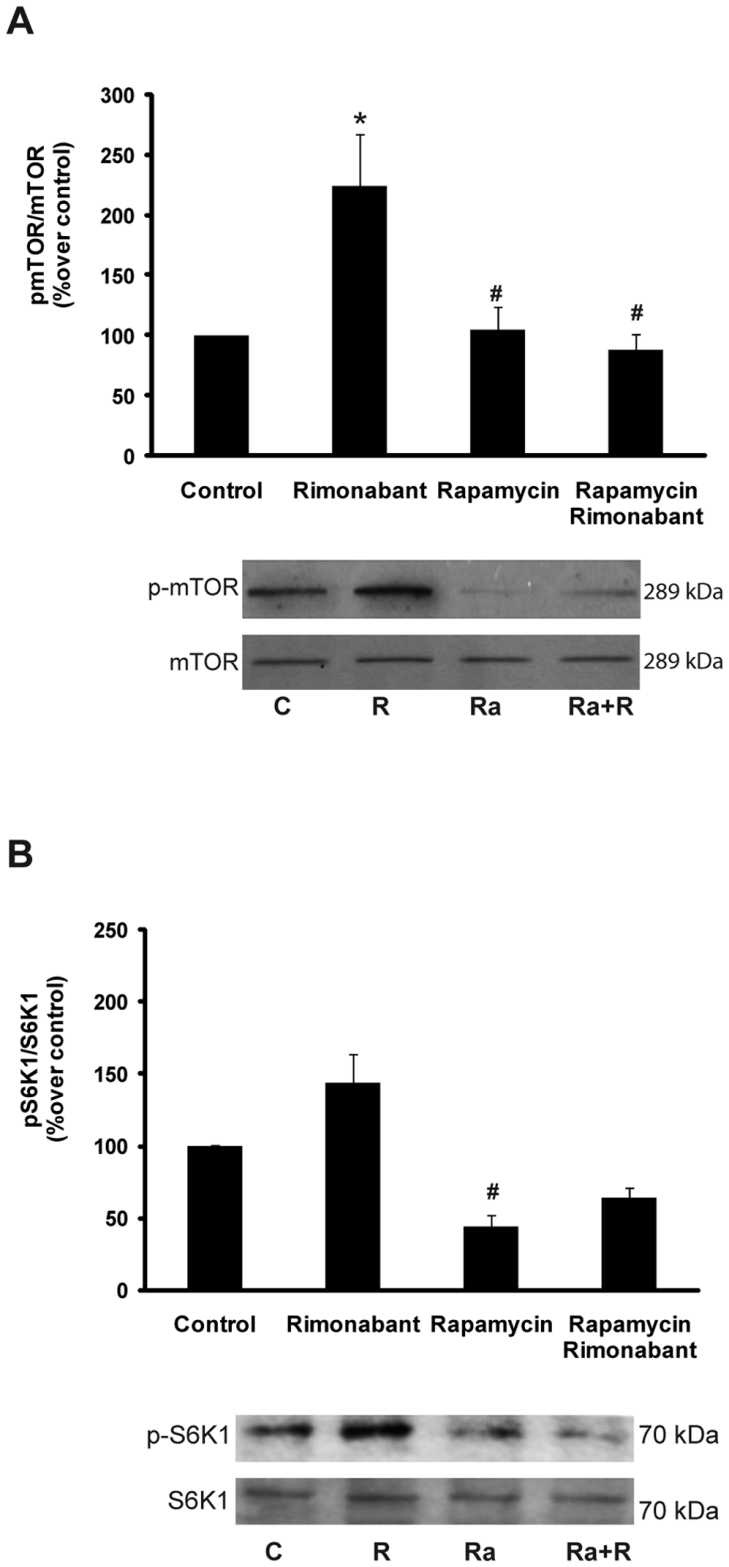
Measures of pmTOR/mTOR levels in gastric mucosa and representative Western blots from animals in the fasting state treated with ip rimonabant and/or ip rapamycin chronically for 1 week (A). Phospho-S6K1/S6K1 in gastric mucosae from animals in the fasting state treated with rimonabant ip and/or rapamycin ip chronically for 1 week (B). The results are expressed as percentages over control (phospho-S6K1/S6K1). *p<0.05, vs control; ^#^p<0.05 vs rimonabant. C: control, R: rimonabant, Ra: rapamycin; Ra+R: rapamycin+rimonabant.

Gastric CB1 blockade by rimonabant treatment in the fasting state induced activation of the mTOR/S6K1 pathway as demonstrated by the pmTOR and mTOR data, which are expressed as percentages over control, (fast: 100 vs rimonabant: 224.58±42.83 pmTOR/mTOR; p<0.05). Additionally, rimonabant treatment was not able to reverse the blockade of the mTOR/S6K1 pathway that was elicited by rapamycin (rapamycin: 104.5±16.92 vs rapamycin+rimonabant: 87.75±13.85 pmTOR/mTOR; Figure 5A).

Gastric phospho-S6K1 responded to rimonabant treatment in a manner similar to that of pmTOR (control: 100 vs. rimonabant: 143.58±6.47 pS6K1/S6K1; no significant difference). Additionally, and in accordance with previous studies, the intracellular pathway was inhibited by rapamycin treatment (control: 100 vs. rapamycin: 43.86±5.20 pS6K1/S6K1), and under this condition, rimonabant did not affect pS6K1 (rapamycin+rimonabant: 64.41±10.13 pS6K1/S6K1) (Figure 5 B). All the values are represented as percentages over control.

### Experiment 6: The effects of pharmacological blockade of CB1 on plasma ghrelin and gastric ghrelin secretion in animals with inhibited gastric mTOR signaling

**Figure 6:**
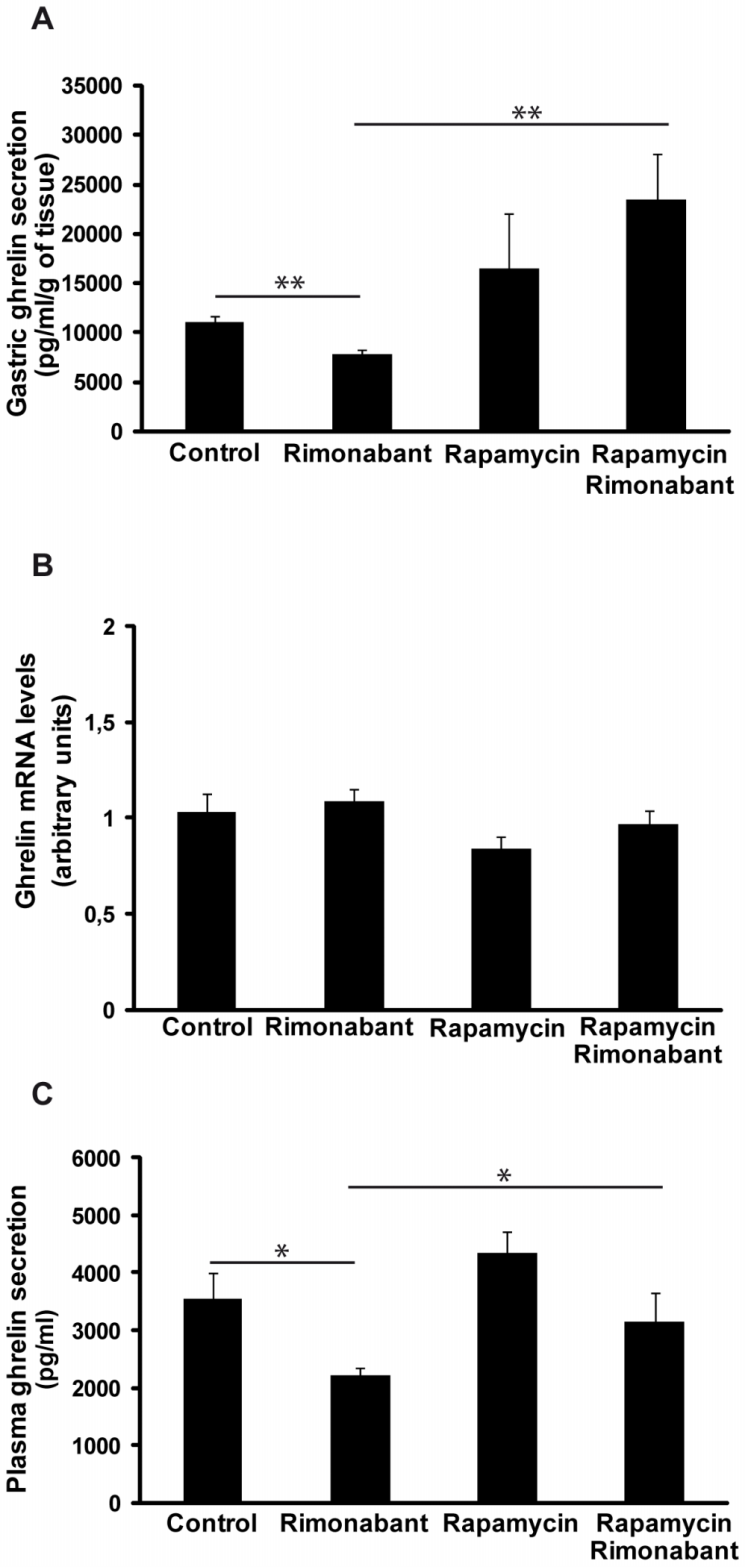
Gastric ghrelin secretion (A). Ghrelin mRNA levels measured by real-time PCR in the gastric mucosae (B) and plasma ghrelin levels (C) from animals in fasting states treated with ip rimonabant and/or ip rapamycin chronically for 1 week.

Blockage of the mTOR pathway with chronic rapamycin treatment blocked the effects of rimonabant on gastric ghrelin secretion (Figure 6A), ghrelin mRNA expression in gastric mucosae (Figure 6B) or circulating ghrelin levels (Figure 6C). However, the groups that received rapamycin treatment exhibited higher levels of gastric ghrelin secretion (Control: 11060±559 vs rapamycin: 16452±5489 pg/ml/g of tissue; rimonabant: 7796±4741 vs rapamycin+rimonabant: 23337±4741 pg/ml/g of tissue, p<0.01). Similarly, plasma ghrelin levels were increased in the animals that received rapamycin treatment (control: 3559±445 vs rapamycin: 4350±375 pg/ml/g of tissue; rimonabant: 2211±141 vs rapamycin+rimonabant: 3160±504 pg/ml/g of tissue, p<0.01).

### Experiment 7: The effects of pharmacological blockade of CB1 with AM281 on plasma ghrelin and gastric ghrelin secretion

To confirm the inhibitory effects of the blockade of the peripheral CB1 receptors on gastric ghrelin secretion from the stomach and plasma ghrelin levels, several measures were performed on samples from fasting animals treated with an alternative CB1 antagonist (AM281).

**Figure 7:**
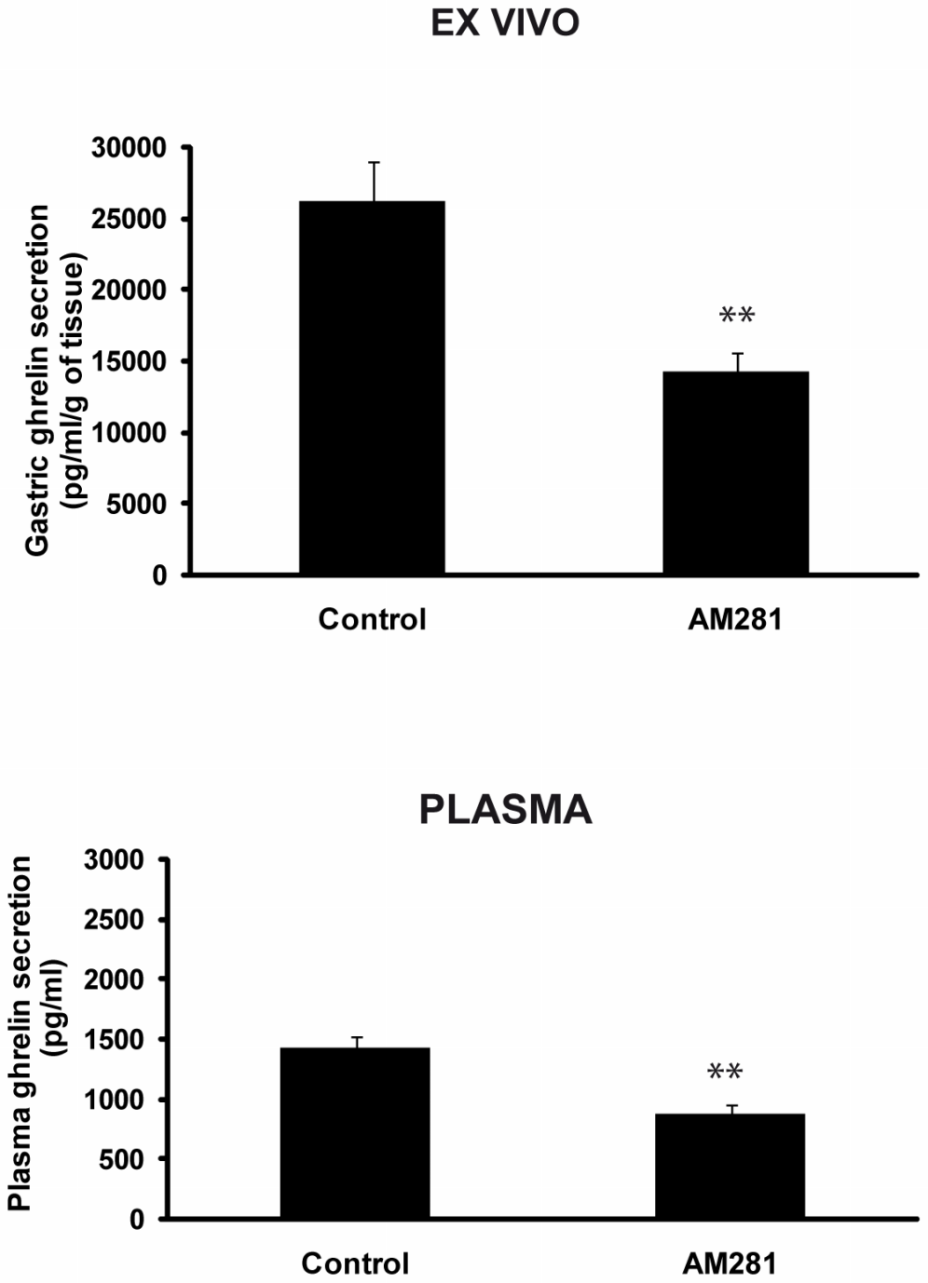
Gastric ghrelin secretion from tissue explants (A) and plasma ghrelin levels (B) obtained from 36-hour fasted animals that received in vivo treatment with AM281 (3 mg/Kg ip). **p<0.01.

AM281 treatment (ip) of fasted animals inhibited the elevated levels of gastric ghrelin secretion induced by food deprivation (fast: 26170±2786 pg/ml/g tissue vs fast AM281: 14222.78±1329.3 pg/ml/g tissue; p<0.01 (Figure 7A).

Corroborating ex vivo data, pharmacological cannabinoid receptor blockade with AM281 prevented food-deprivation-induced increases in circulating ghrelin levels (fast: 1424±99.96 pg/ml vs fast rimonabant: 876±67.25 pg/ml; p<0.01) (Figure 7B).

### Experiment 8: The effect of pharmacological blockade of CB1 with AM281 on the regulation of gastric mTOR signaling

**Figure 8:**
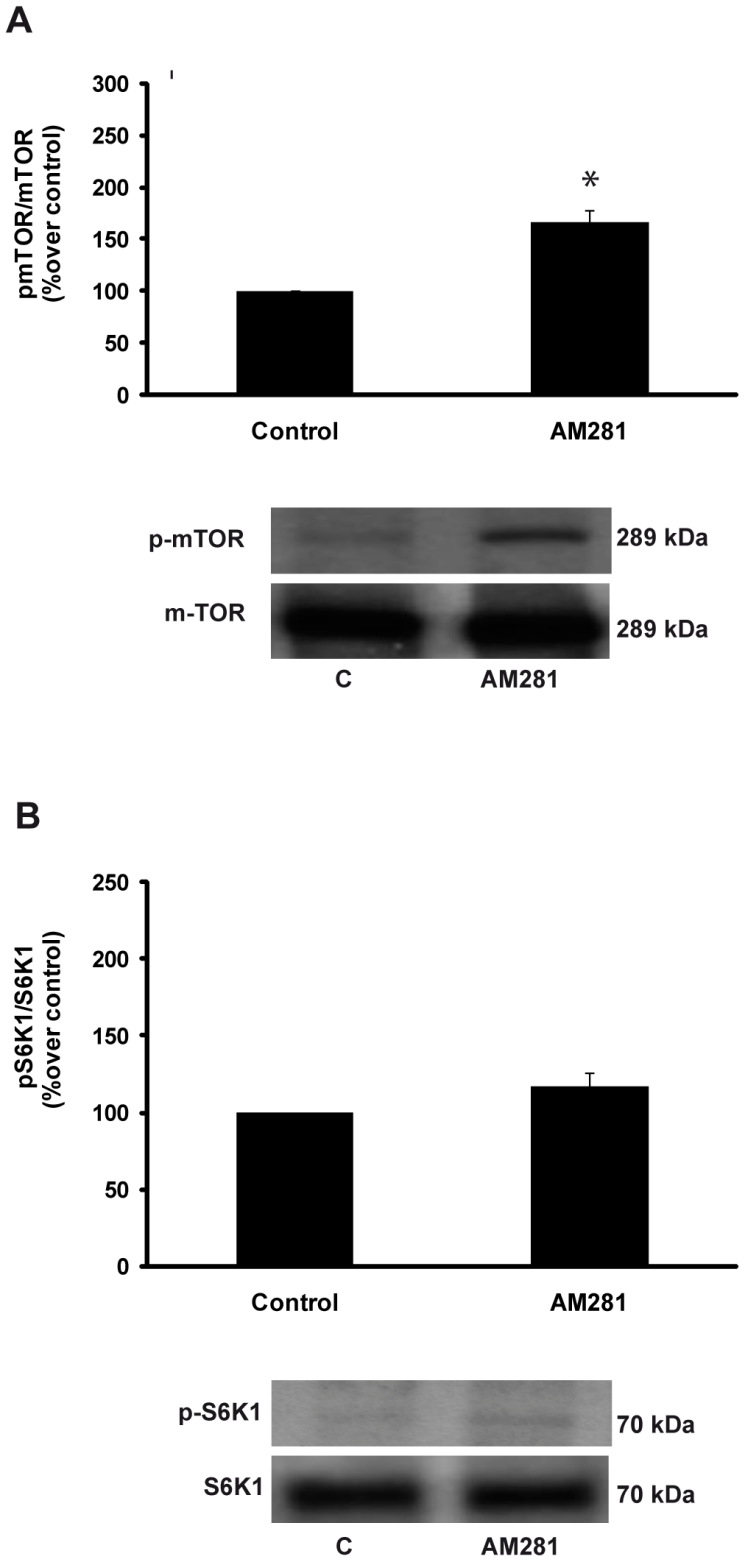
Measures of pmTOR/mTOR in the gastric mucosae and representative Western blots from animals in the fasting state treated with ip AM281 or vehicle (A). Phospho-S6K1/S6K1 in the gastric mucosae and representative Western blots from animals in the fasting state treated with ip AM281 or vehicle (control) (B). The results are expressed as percentage over control (phospho-S6K1/S6K1). *p<0.05.

Corroborating the effects of rimonabant, gastric CB1 blockade wit AM281 treatment in the fasting state induced activation of the mTOR/S6K1 pathway as demonstrated by the pmTOR and mTOR data, which are expressed as percentages over control, (control: 100 vs. AM281: 166.72±10.66 pmTOR/mTOR; p<0.05, Figure 8A).

As rimonabant treatment, gastric phospho-S6K1 responded to AM281 treatment in a manner similar to that pmTOR, although the phospho-S6K1 response did not reach statistical significance (control: 100 vs AM281: 116.71±9.04 pS6K1/S6K1; no significant difference, Figure 8B). All values are represented as the percentages over control.

## Discussion

The main findings of the present work are as follows: 1) peripheral CB1 receptors modulate food intake through a mechanism that implies gastric ghrelin regulation that requires intact vagal communication; 2) CB1 receptors are localized in the neuroendocrine cells of the stomach; and 3) Gastric CB1 receptors modulate gastric ghrelin secretion at the intracellular level through the mTOR pathway.

Previous studies have suggested that the endocannabinoid system is involved in appetite and feeding [22], and multiple experimental protocols have demonstrated the food intake-reducing effects of rimonabant, which is an antagonist of the CB1 receptor. Accordingly, in the present work, peripherally injected rimonabant was found to prevent the increase in food intake elicited by central ghrelin administration (Figure 1A and B). Moreover, a more surprising finding was that rimonabant *perse* was able to induce a reduction in basal food intake amounts but only in the animals that were fasted overnight (Figure 1B). However, the anorexigenic effects of rimonabant disappeared if the animals had previously been subjected to surgical vagotomy (Figure 1C); this finding highlight the requeriment of an intact vagus connection for the effects of the cannabinoid system on appetite. This finding has previously been suggested by the work of Gomez et al., who demonstrated that systemic capsaicin treatment, which selectively destroys unmyelinated visceral afferents, blocks the inhibitory effect of rimonabant [13]. However, another study from Madsen et al. demonstrated that intact vagal afferents are not required for the anorexigenic action of rimonabant [23]. The main difference between the work of Madsen et al. and the present study was the administration route. In the previous study, rimonabant was administered via oral gavage, whereas in our work and the work of Gomez rimonabant was administered intraperitoneally.

It has been repeatedly shown that animals in fasting states exhibit increased levels of ghrelin that induce orexigenic effects mainly via the activation of the vagus nerve [24], [25], and the present study supports the possibility that peripheral rimonabant acts locally at the gastric level to reduce ghrelin secretion and consequently, downregulate food intake. The immunohistochemical studies performed in the present work showed that the neuroendocrine cells producers of ghrelin are distributed near the CB1-positive cells in the stomach and have patterns of distribution that are similar to those of the CB1-positive cells (Figure 2). CB1 expression in rats has been described in pre and postganglionic cholinergic neural elements that innervate the smooth muscle and mucosal and submucosal blood vessels [26]. However, these studies did not demonstrate the localization of these receptors to the neuroendocrine cells of the stomach.

After finding an anorexigenic effect of rimonabant on basal and ghrelin-stimulated food intake and immunopositivity for CB1 and ghrelin in the neuroendocrine cells of the stomach, we next determined whether the interaction between these systems was mediated locally in the stomach. To do this, we used an organ culture model of gastric tissue that was previously developed and validated by our group [19], [20], [25], [27]. With this model, peripherally administered rimonabant was shown to reverse the gastric increase in ghrelin secretion induced by fasting (Figure 3B). However, in fed animals, which exhibit basal levels of ghrelin secretion, the administration of rimonabant failed to produce any effect (Figure 3A). The present data support the results obtained from the food intake studies in which the administration of rimonabant to fed animals did not produce an anorexigenic effects (Figure 1A), but did produce strong effects in overnight fasted animals (Figure 1B). In the light of this finding, we can affirm that the modulation of gastric ghrelin secretion by CB1 is required for the anorexigenic effect of antagonists of this receptor. Moreover, in accordance with the lack of effect of rimonabant on food intake in vagotomized animals (Figure 1C), the gastric studies of the present work indicate that the inhibition of ghrelin by rimonabant was abolished after vagotomy (Figure 3B), which, in turn, indicates that the vagus connection needs to be intact to ensure efficient interactions between ghrelin and CB1. Consequently, in the in vitro experiment in which, gastric tissues received one rimonabant treatment in isolation from the complete organism (i.e., the vagus enervation was not fully operational), inhibition of ghrelin by rimonabant was not observed (Figure 3E and F). To corroborate these results and to assess whether the secretion of ghrelin by the stomach is reflected in circulating ghrelin levels, the plasma ghrelin levels of the experimental groups were measured. As shown in the Results section (Figure 3D and 7B), the peripheral antagonism of CB1 was reflected by a decrease in circulating ghrelin levels, which was a consequence of the reduction of ghrelin secretion from the stomachs of the fasted animals. These data corroborate the findings of other authors who have described the regulation of ghrelin plasma levels by peripheral treatment with different agonists or antagonist of the CB1 receptor [28], [29].

The studies of the secretion, protein contents and mRNA levels of ghrelin in gastric tissue allowed us to establish a gastric mechanism for the regulation of ghrelin by cannabinoids. The above mentioned mechanism was clearly demonstrated by the decrease in ghrelin secretion from the stomach (Figure 3B) that was induced by rimonabant ip treatment; this reduction in ghrelin secretion was accompanied by increased amounts of protein storage in the ghrelin-positive cells (Figure 4D), as measured by Western blot, which was accompanied by no effect on ghrelin mRNA levels (Figure 4B). Moreover, an increase in gastric ghrelin secretion by the fasted animals compared to the *ad libitum* fed group was observed (Figure 3A and B); this finding was reflected by the reduced ghrelin content inside the cells as shown by Western blots (Figure 4E and F). Together, the data presented in the present work reinforce the physiological mechanism of the regulation of gastric ghrelin secretion that has previously been reported in other conditions by our group [20].

The CB1 mRNA expression levels in the gastric mucosae were assayed with real-time PCR in the experimental models, and, to us, the most interesting findings was the significant decrease in CB1 mRNA levels in the stomachs of fasted animals after peripheral rimonabant treatment and the lack of an effect in the fed animals (Figure 4E and F). These data reinforce the finding that the effects of rimonabant on ghrelin and the regulation of food intake are observed only in fasted animals. The rimonabant induced downregulation of CB1 might be associated with the decreases in ghrelin secretion by the stomach [11]. In contrast, surgical vagotomy induced decreases in CB1 receptors in both fed and fasted animals, which might explain the lack of effect of rimonabant on food intake in the animals subjected to surgical vagotomy (Figure 1C).

The second part of this study focused on the search for the intracellular mechanism responsible for the interaction between ghrelin and the endocannabinoid system in the stomach. The possible role of the mTOR pathway in mediating the gastric interaction between ghrelin and endocannabinoid system was explored. To the best of our knowledge, our study is the first to demonstrate that the acute administration of rimonabant or AM281 to fasted animals activates the mTOR/S6K1 pathway as demonstrated by the increases in the phosphorylation of mTOR (Figure 5A and 8A) and the non-significant trend towards increased phophorylation of S6K1, a downstream effector of mTOR (Figure 5B and 8B). In accordance with our results, it has previously been described that mTOR phosphorylation in the gastric mucosa is downregulated by fasting, and we demonstrated that this effect was reverted after rimonabant administration (Figure 5A). It has previously been reported that the inhibition of gastric mTOR signaling leads to increased expressions of ghrelin mRNA, GOAT mRNA, tissue preproghrelin content and circulating ghrelin and that there is an inverse relationship between gastric mTOR signaling and ghrelin expression and secretion during changes in energy status [30]. Accordingly, in the present paper, the decreases in gastric ghrelin secretion (Figure 3B and 7A) and circulating ghrelin (Figure 3D and 7B) levels that were observed in the fasted animals treated with rimonabant or AM281 coincided with increase in mTOR and S6K1 phosphorylation (Figure 5A and B and Figure 8A and B).

Finally, we confirmed our results using an in vivo model in which the mTOR/S6K1 pathway was blocked with the objective of proving the key role of this intracellular pathway in this gastric system. To do this, the effects of the administration of rimonabant to fasting animals that had received chronic rapamycin treatment on gastric and plasma ghrelin levels were tested. Rimonabant treatment did not affect mTOR or S6K1 phosphorylation in animals that has been treated with rapamycin (Figure 5A and B). Moreover, the injection of rimonabant into the fasted animals whose mTOR pathways had been blocked by rapamycin treatment did not affect gastric ghrelin secretion (Figure 6A), ghrelin mRNA (Figure 6B) or circulating ghrelin levels (Figure 6C), thus demonstrating that the mTOR pathway is responsible for the interaction between the endocannabinoid system and ghrelin at the gastric level. However, the involvement of additional gastric-level signaling routes in this system should not be excluded and the futures studies should focused on identifying additional intracellular pathways involved in this system of gastric regulation.

In summary, to the best of our knowledge, these data demonstrate for the first time that pharmacological blockade of the cannabinoid receptor via peripheral rimonabant treatment is sensed by gastric cells as a satiety signal that is comparable to food intake, as shown by the activation of the mTOR/S6K1 pathway. Consequently, the gastric tissue responds by inhibiting the increased ghrelin secretion that is normally observed in fasting states, which results in decreased food intake due to decreased vagally mediated orexigenic signaling to the brain.

In conclusion, the present work demonstrates the existence of a gastric mechanism for the interaction between the endogenous cannabinoid system (CB1) and ghrelin that is mediated throught the intracellular mTOR pathway and, results in a physiological effect on the regulation of food intake that requires the vagus nerve. This paper used molecular, physiological and surgical approaches to highlights the metabolic action of the gastric tissue that emerges as a result of the complex network of relevant endocrine systems. Additionally these data provide new clues for the understanding of the complex mechanisms of the regulation of food intake.

## Supporting Information

Figure S1 Gastric ghrelin secretion from tissue explants from 36-hour fasted animals that received different in vitro treatments: vehicle (control), rimonabant (2.5 µM) or rimonabant (5 µM).
(TIF)
