# Supplementary figures and images for: The Gastric CB1 Receptor Modulates Ghrelin Production through the mTOR Pathway to Regulate Food Intake
Source: PLoS One. 2013 Nov 26;8(11):e80339. doi: 10.1371/journal.pone.0080339 (PMC3841176; doi:10.1371/journal.pone.0080339)

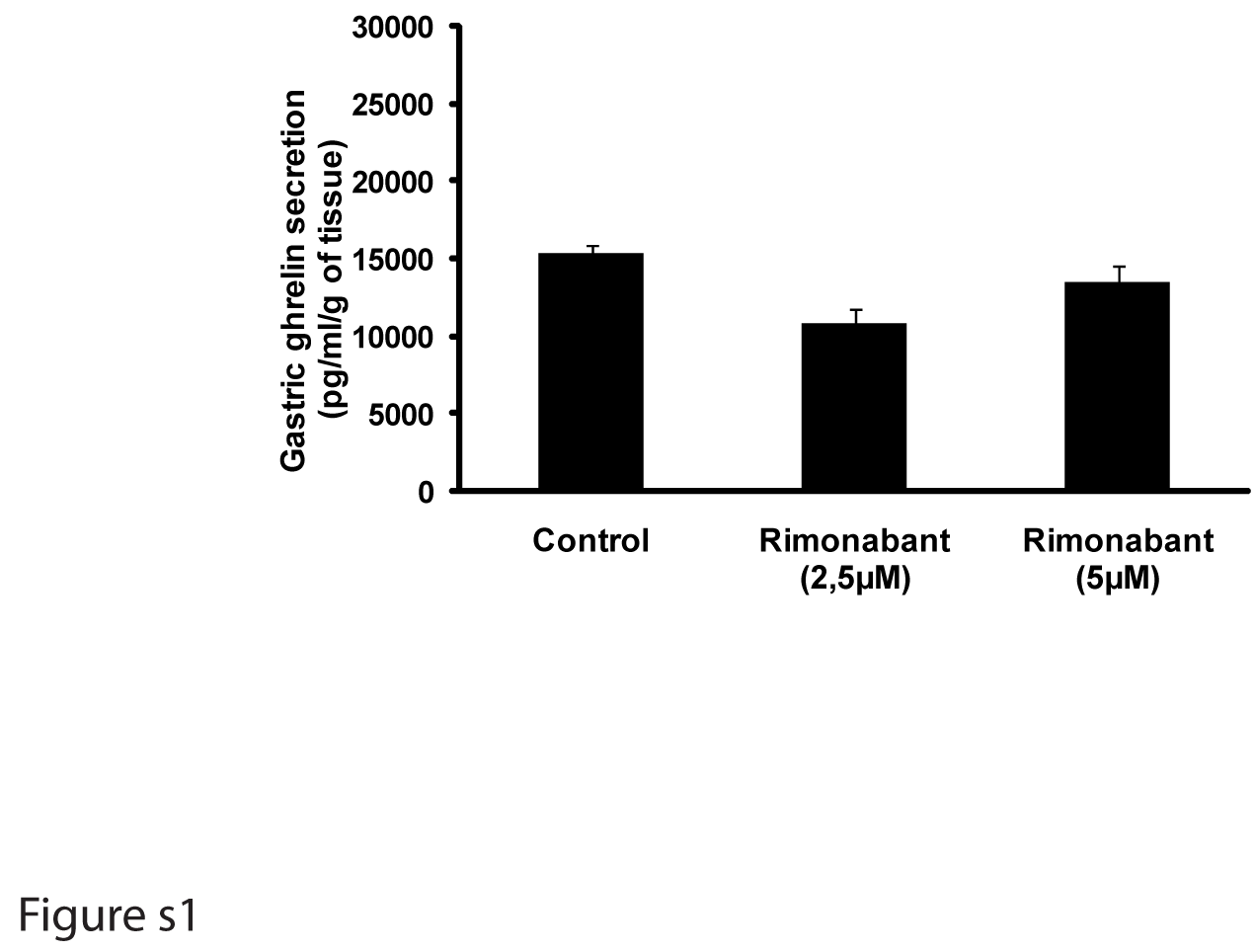

Supplement: Figure S1 — Gastric ghrelin secretion from tissue explants from 36-hour fasted animals that received different in vitro treatments: vehicle (control), rimonabant (2.5 µM) or rimonabant (5 µM). (TIF) [file pone.0080339.s001.tif]
